# Corrective wedge ostectomy for an atypical femoral procurvatum deformity stabilised with a supracondylar bone plate

**DOI:** 10.4102/jsava.v89i0.1545

**Published:** 2018-12-05

**Authors:** Tesh M. Smalle, Gert L. Coetzee, Stephanus H. Naude

**Affiliations:** 1 Translational Research and Animal Clinical Trial Study Group (TRACTS), U-Vet Veterinary Teaching Hospital, University of Melbourne, Australia; 2 Department of Companion Animal Clinical Studies, University of Pretoria, South Africa; 3 Johannesburg Specialist Veterinary Centre, Johannesburg, South Africa

## Abstract

Physeal fractures of the distal femur are among the most commonly encountered fractures in skeletally immature dogs. These fractures respond poorly to conservative management and thus early surgical reduction and stabilisation are recommended. A 7-month-old intact male Border collie presented with a history of chronic lameness. Clinical examination revealed a predominantly non-weight-bearing lameness of the right hindlimb and concurrent muscle atrophy. A pronounced, but atypical, procurvatum deformity of the right distal femur was diagnosed on survey radiographs. Malunion of a Salter–Harris Type III physeal fracture was suspected as there was an associated history of trauma. A cranially based closing wedge ostectomy was performed to address the femoral deformity and subsequently stabilised using a supracondylar bone plate. The dog recovered well and was moderately weight-bearing lame on the right hindlimb 6 weeks post-operatively. Ten months following the operation the range of motion had improved in the right stifle and no signs of lameness were evident at a walk. We advocate surgical correction of sagittal plane deformities of the distal femur using the CORA method. Overall, a good functional outcome was achieved, which is consistent with previously reported cases with similar deformities.

## Introduction

Physeal fractures of the distal femur are among the most commonly encountered fractures in skeletally immature dogs (Kim & Lewis 2014). These fractures respond poorly to conservative management. Early surgical reduction and stabilisation is recommended to prevent further physeal injury and malunion of these fractures (Fox & Tomlinson 2012; Kim & Lewis 2014; Kuipers von Lande & Worth 2010; Manfra Maretta & Schrader 1983).

‘Malunion’ is defined as a healed fracture where the anatomical alignment of the bone was not achieved or maintained during the healing process (Fox & Tomlinson 2012; Kuipers von Lande & Worth 2010). Malunion of distal femoral physeal fractures usually results in a pronounced procurvatum deformity of the distal femur because of the tension applied to the caudal aspect of the tibia by the semi-tendinosus and semi-membranosus muscles, with caudal traction of the distal femoral epiphysis through the cruciate ligaments. This deformity results in an inability to fully extend the affected stifle joint as the quadriceps mechanism is impeded. Additionally, contracture of the hamstring muscles and fibrosis of the joint capsule, resulting from disuse of the affected hind limb, further hinders stifle extension. Secondary degenerative joint disease ensues (Kim & Lewis 2014).

Angular limb deformities are classified according to the direction of the angulation in a specific plane, the number of centres of rotation of angulation and their directional relationship (Fox & Tomlinson 2012). Surgical management of cases with various angular limb deformities resulting from malunited distal femoral physeal fractures are infrequently described. Their management involves corrective osteotomies and surgical stabilisation using hybrid and/or dynamic external skeletal fixators, reconstruction plates, String of Pearls locking plates or supracondylar bone plates (Coutlin et al. 2013; Kim & Lewis 2014; Kuipers von Lande & Worth 2010; Lewis et al. 1993; Petazzoni & Palmer 2012; Roch & Gemmill 2008). The surgical technique can be challenging as the small size and natural procurvatum of the distal fragment limit the amount of bone available for appropriate fixation (Kim & Lewis 2014).

This case report documents an atypical procurvatum deformity of the distal femur caused by malunion of a suspected Salter–Harris Type III fracture of the distal femoral physis. It documents the surgical planning, execution and stabilisation of the corrective wedge ostectomy using a supracondylar bone plate.

## Case presentation

A 7-month-old, 14.8 kg, intact male Border collie was presented at the Onderstepoort Veterinary Academic Hospital with a history of chronic lameness. The owner reported that a traumatic event may have been associated with the onset of this lameness. On clinical examination, a predominantly non-weight-bearing lameness of the right hindlimb was confirmed. Additionally, moderate muscle atrophy of predominantly the quadriceps muscle group was evident on the right (mid-femoral circumference of 30.5 centimetres (cm) vs. 33.5 cm on the left). Subjectively, when the dog was placed in dorsal recumbency with hip and stifle joints flexed to 90 degrees (°) bilaterally, the right femur was markedly shorter than the left femur. Full extension of the right stifle joint was not possible with the range of motion being limited to 54° in flexion and 115° in extension (normal range of motion of the stifle joint in Labrador retrievers is 41° in flexion and 162° in extension (Jaegger, Marcellin-Little & Levine 2002). Crepitus was palpable over the patella on manipulation of the right stifle. The patella could not be luxated and no instability was evident on palpation of the stifle joint.

Radiography of the affected and contralateral femur was performed. A true craniocaudal radiograph of the affected right femur could not be obtained because of an inability to fully extend the stifle joint. This resulted in a foreshortened radiograph of the right femur. A true craniocaudal radiograph of the femur is where the patella is situated in the centre of the distal femur, the lateral and medial fabella are bisected by their respective femoral cortices and there is slight protrusion of the lesser trochanter (Dismukes et al. 2008). Mediolateral radiographs were obtained with the condyles superimposed. A marker was placed at the height of the femoral shaft as a calibration device.

**FIGURE 1:**
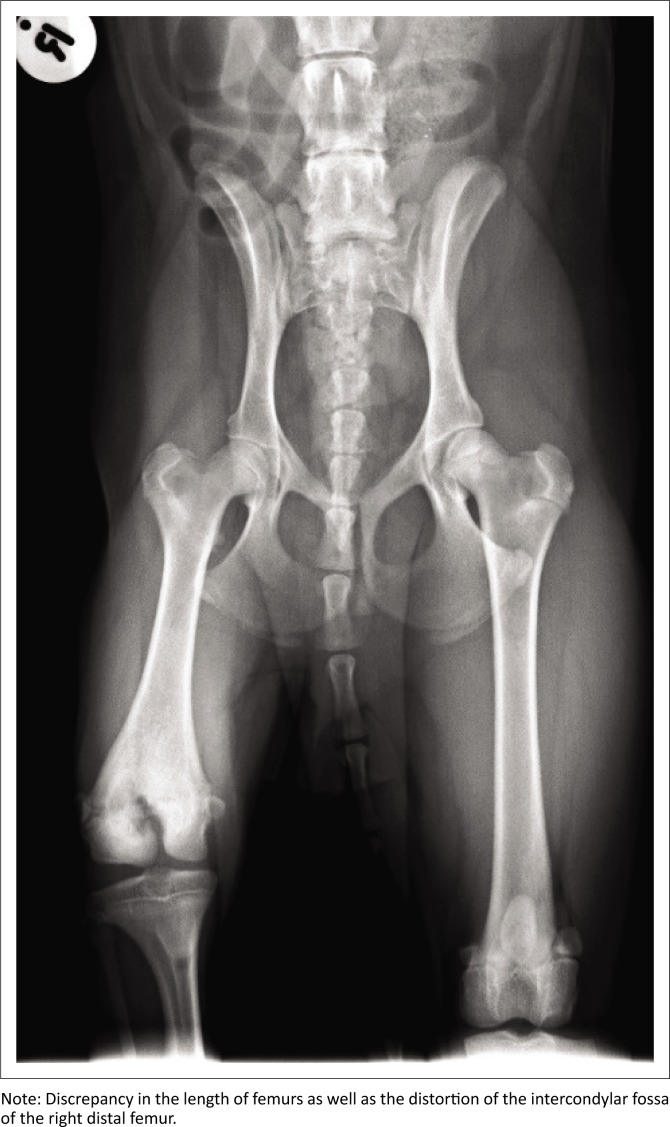
Radiographic image: Preoperative ventrodorsal view of the pelvis.

Radiographs revealed an atypical procurvatum deformity that was centred over the right distal femoral physis. The mediolateral view of the right femur showed distal metaphyseal flaring, with the right distal metaphysis measuring 33% wider than the left. Additionally, there was sclerosis of the caudal aspect of the right distal metaphysis and a solid periosteal reaction over the cranial mid to distal diaphysis. The right distal femoral physis was indistinct and distorted caudally. Mediolateral views of the right femur showed that it was 18% shorter than the left. Additionally, the intercondylar fossa of the right distal femur was distorted and measured 19 millimetres (mm) deep. The medial condyle of the right distal femur appeared to show signs of hypoplasia (Figure 1). An accurate evaluation of a frontal plane deformity was not possible as a true craniocaudal radiograph of the affected femur was not obtained. A torsional deformity of the affected femur was not suspected as the patella could not be luxated. Obvious joint effusion was present in the right stifle with displacement of the infrapatellar fat pad. The muscular atrophy was confirmed on radiographs.

**FIGURE 2:**
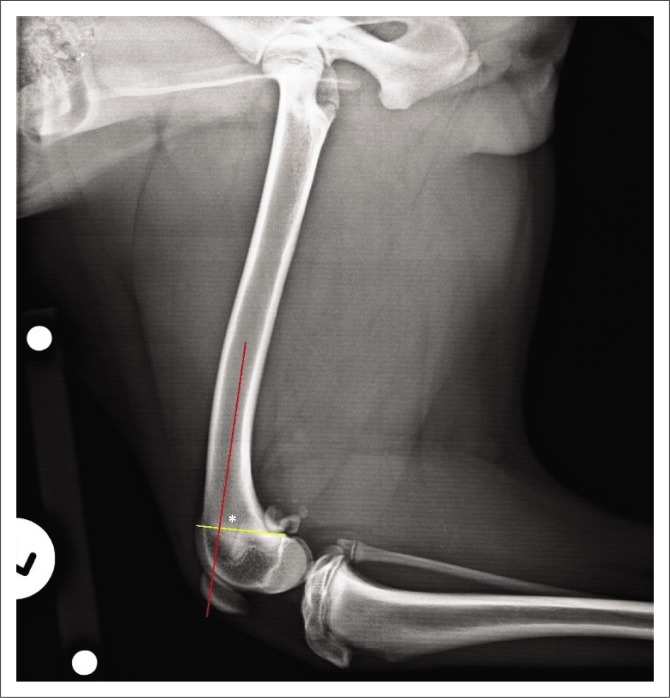
Radiographic image: Preoperative mediolateral view of the left femur. The anatomical caudal distal femoral angle (*) was calculated as 86° on the left.

**FIGURE 3:**
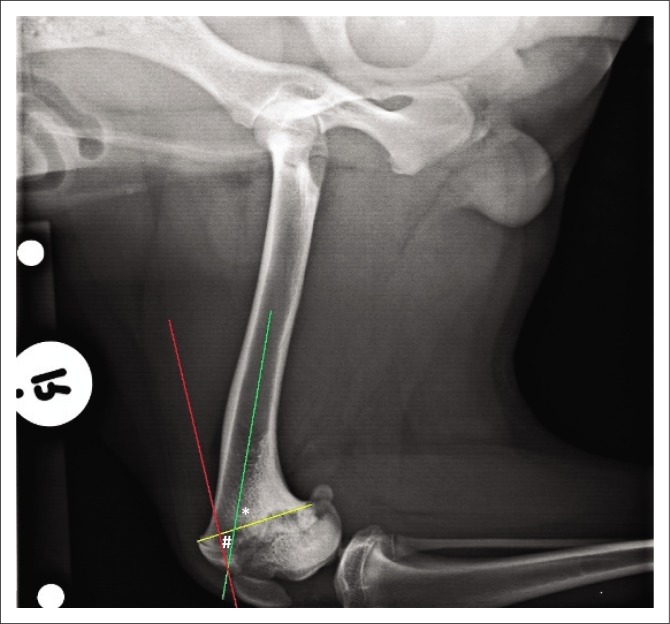
Radiographic image: Preoperative mediolateral view of the right femur. The centre of rotation of angulation (#) measured 23° and was located at the most cranial extent of the right distal femoral epiphysis.

The centre of rotation of angulation (CORA) method was used to define the sagittal plane deformity and plan the corrective osteotomy from the mediolateral radiographs (Fox & Tomlinson 2012; Kara et al. 2018; Kim & Lewis 2014; Raske et al. 2013). First, the distal femoral joint orientation line (yellow line) was defined as a line connecting the points where the distal femoral physeal scar exited cranially and caudally. Next, the anatomical axis of the distal femur (red line in Figure 2, green line in Figure 3) was defined as the line bisecting the distal diaphysis and metaphysis of each femur. This allowed the anatomical caudal distal femoral angle (aCdDFA) to be calculated as the angle formed between the distal femoral joint orientation line and anatomical axis (Figure 2). The aCdDFA was calculated as 86° on the left and 63° on the right.

**FIGURE 4:**
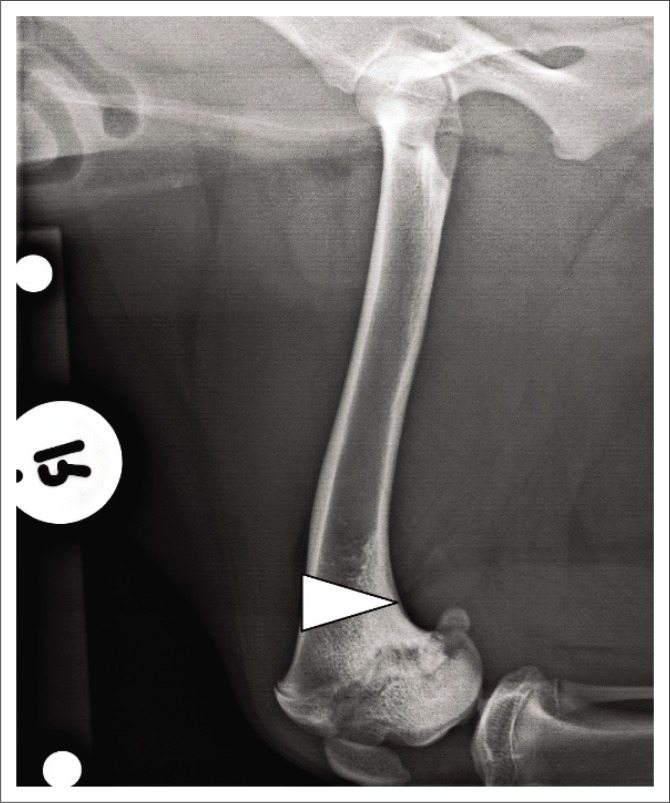
Radiographic image: Preoperative mediolateral view of the right femur. A 23° cranially based closing wedge ostectomy was planned.

To define the CORA of the deformity, the anatomical axis of the normal left distal femur (red line) was placed at an angle of 86° to the distal femoral joint orientation line (yellow line) of the abnormal right femur. Careful measurement ensured that the anatomical axis of the left distal femur intersected the distal femoral joint orientation line at the same distance caudal to the cranial exit of the physeal scar on both femurs. The CORA of the deformity was defined as the location where the distal femoral anatomical axes (red line and green line) intersected each other when superimposed on the abnormal right femur (Figure 3). In this case, it was located at the most cranial aspect of the right distal femoral epiphysis and measured 23°. The transverse bisecting line and transverse angles were not calculated as the CORA was located too far distally to allow the corrective osteotomy to be performed at this level. A cranially based closing wedge of 23° was planned in this case (Figure 4). The base of the triangle was situated over the cranial cortex and measured 1 cm. The apex converged on the caudal cortex of the distal femur.

The patient was premedicated with acepromazine hydrochloride (Aceprom, Bayer, Isando, South Africa) at 0.01 milligrams per kilogram (mg/kg) subcutaneous (SC) and morphine (morphine sulphate, Fresenius PF, Port Elizabeth, South Africa) at 0.4 mg/kg SC approximately 30 minutes (min) prior to induction. Anaesthesia was induced using propofol (Propofol Fresenius vial, Fresenius Kabi, Midrand, South Africa) at 6 mg/kg intravenous (IV) to effect. An endotracheal tube was placed to allow anaesthetic maintenance with isoflurane (Forane, Abbott Laboratories, Johannesburg, South Africa). Cefazolin sulphate (Zefkol 1 g injection, Aspen Pharmacare, Durban, South Africa) was administered at 22 mg/kg IV 30 min preoperatively, then every 90 min intraoperatively. Morphine (morphine sulphate, Fresenius PF) was continued at 0.3 mg/kg IV every 120 min intraoperatively. An epidural block was aseptically performed by administering morphine (morphine sulphate, Fresenius PF) at 0.2 mg/kg and ropivacaine at 1 mg/kg (Naropin; AstraZeneca Pharmaceuticals, Johannesburg, South Africa) into the lumbosacral epidural space. The patient was aseptically prepared for surgery and placed in left lateral recumbency. A routine surgical approach was performed to the right distal femur and stifle joint through a lateral incision (Paatsama 2004). Exploration of the stifle joint revealed a shallow trochlear groove with fibrous adhesions to the trochlear sulcus and osteophytes on the lateral and medial trochlear ridges. The cranial cruciate ligament was intact. A cranially based closing wedge ostectomy was planned using an oscillating saw. Two 2.0 mm Kirschner wires were placed as alignment aids in a craniocaudal direction, with one on either side of the proposed osteotomy site to allow a more orthogonal osteotomy to be performed (Addison et al. 2015). The level of the wedge ostectomy was such that it allowed an adequate size of the distal fragment for placement of a 3.5-mm supracondylar bone plate. Preoperative planning dictated that the cranial base of the wedge was 10 mm and the apex converged on the caudal aspect of the distal femur. The wedge ostectomy margins were reduced and stabilised with three divergent 1.6-mm Kirschner wires placed in a normograde fashion. Once stabilised a 3.5-mm supracondylar bone plate was contoured to the lateral aspect of the distal femur. Four bicortical screws were placed in the proximal fragment. One bicortical and three monocortical screws were placed in the distal fragment. Placement of bicortical screws in the distal fragment was hindered by the distorted anatomy of the intercondylar fossa and fear of iatrogenic damage to the cruciate ligaments. Screws were angulated to avoid the ostectomy site. A trochlear block recession was performed (Fox & Tomlinson 2012), followed by routine closure of the surgical site.

Post-operative radiographs revealed adequate implant positioning. Determination of the post-operative aCdDFA was not possible as the implants obscured the necessary landmarks. Subjectively, the operated limb was still markedly shorter but not worse than preoperatively.

The patient was admitted to an intensive care unit and maintained on intravenous Ringer’s lactate solution (Sabax Ringer’s lactate, Adcock Ingram Critical Care, Johannesburg, South Africa) for 48 h. The operated limb was placed in a protective bandage for 96 hours (h). The analgesic protocol included morphine (morphine sulphate, Fresenius PF) administered at 0.2 mg/kg – 0.4 mg/kg IV every 4 h for 48 h. Additionally, firocoxib (Previcox, Merial, Midrand, South Africa) was administered at 5 mg/kg per os PO (by month, once daily) for 10 days. Cephazolin (Zefkol 1 g injection, Aspen Pharmacare, Durban, South Africa) was administered at 22 mg/kg IV bid for 24 h and then cephalexin (Ranceph, Rabaxy SA [Pty] Ltd, Centurion, South Africa) was continued at 20 mg/kg PO twice daily for a further 5 days. The patient was discharged 1 week later with instructions to restrict exercise, allowing only short leash-walks, and to continue passive range of motion exercises. At this time, the patient was placing the paw with each step but not bearing full weight. Moderate weight-bearing lameness continued in the recovery period.

**FIGURE 5:**
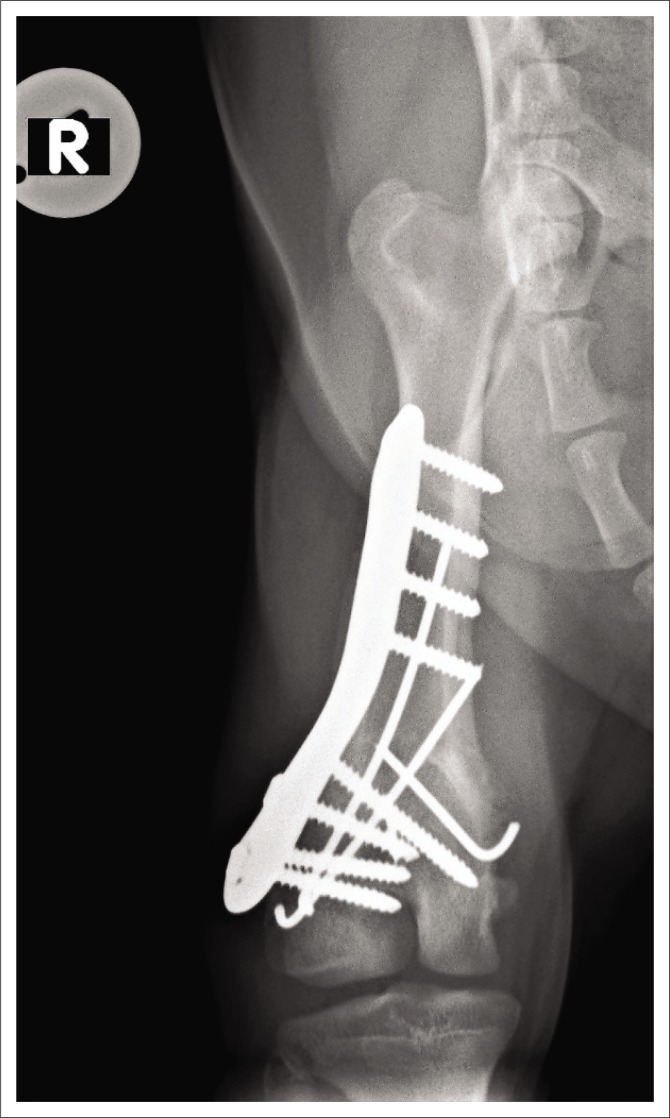
Radiographic image: Follow-up craniocaudal view of the right femur obtained 6 weeks post-operatively.

**FIGURE 6:**
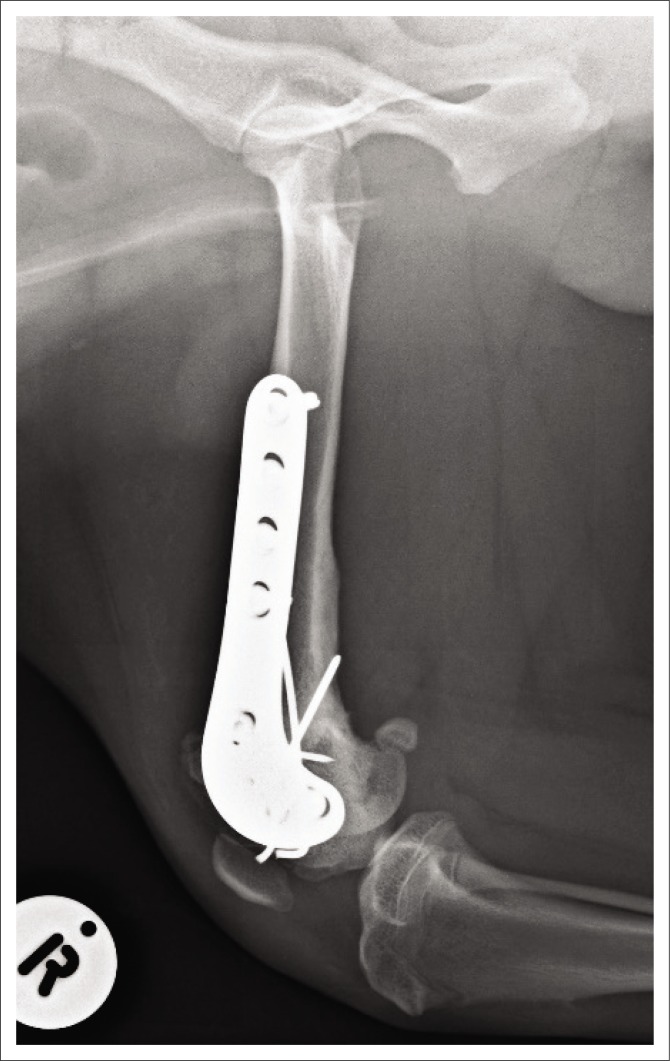
Radiographic image: Follow-up mediolateral view of the right femur obtained 6 weeks post-operatively.

Follow-up radiographs 6 weeks after the operation showed good healing of the ostectomy site with all surgical implants in their original position (Figures 5 and 6). The patient was consistently bearing more weight on the operated limb when compared to preoperative use. Additionally, approximately 50° of motion was gained in stifle extension, but approximately 35° was lost in flexion when compared to preoperative values. At this stage, the activity level was gradually allowed to increase and more rigorous physiotherapy was administered.

At follow-up evaluation 10 months post-operatively, no clinical lameness was evident on the right hindlimb at a walk, and the range of motion of the right stifle had stayed constant when compared to the values determined at follow-up evaluation 6 weeks post-operatively. Obvious crepitus was still palpable over the patella. Radiographs revealed that the ostectomy site had healed fully and that joint effusion had subsided in comparison to preoperative views.

## Discussion

Malunion of distal femoral physeal fractures can result in complex deformities of the distal femur requiring technically challenging corrective osteotomies. Surgical correction of these deformities is infrequently reported but is of paramount importance in correcting the femoropatellar and femorotibial joint incongruity (DeTora & Boudrieau 2016; Kim & Lewis 2014; Kuipers von Lande & Worth 2010; Lewis et al. 1993; Petazzoni & Palmer 2012; Roch & Gemmill 2008).

Similar to previous reports, the caudoproximal displacement of the femoral condyles in this patient resulted in an inability to fully extend the affected stifle joint. This loss of stifle extension can lead to persistent lameness (Kim & Lewis 2014). Interestingly, this displacement of the condyles may have been associated with a malunion of a Salter–Harris Type III fracture of the distal femoral physis. This was suspected as there was an associated history of trauma in this case. The CORA was situated in the most cranial aspect of the distal femoral epiphysis, which may represent the position of a previous fracture. This injury resulted in an atypical procurvatum deformity of the distal femur, as the location of the CORA was in the distal epiphysis and not in the metaphysis as is more commonly reported (Kim & Lewis 2014; Kuipers von Lande & Worth 2010).

Concurrent deformities seen in reported cases include femoral shortening, patellar luxation associated with frontal plane deformities and transverse plane deformities resulting in femoral torsion (DeTora & Boudrieau 2016; Fox & Tomlinson 2012; Kim & Lewis 2014; Kuipers von Lande & Worth 2010; Lewis et al. 1993; Petazzoni & Palmer 2012; Roch & Gemmill 2008). Careful preoperative planning dictated that there would be no significant change in femoral length following completion of the corrective ostectomy. Additionally, according to the literature there is a tendency for the functional length of the affected femur to improve following completion of corrective osteotomies for this type of deformity (Kim & Lewis 2014). Thus, it was decided not to address this discrepancy in femoral length as short-term results show that dogs are able to withstand up to a 20% loss of femoral length in experimental studies (Kim & Lewis 2014; Petazzoni & Palmer 2012). Should this discrepancy become clinically relevant, lengthening of the right femur using distraction osteogenesis or surgical shortening of the left femur can be considered once skeletal maturity is reached (Petazzoni & Palmer 2012).

A multitude of information exists when one is confronted with a frontal plane deformity of the femur. Normal breed reference values have been established and are clinically useful for the diagnosis, treatment and assessment of frontal plane deformities (DeTora & Boudrieau 2016; Fox & Tomlinson 2012; Petazzoni & Palmer 2012; Swiderski & Palmer 2007; Tomlinson et al. 2007). Normal breed reference values and methods for calculating femoral correction in the sagittal plane are not well documented (Kara et al. 2018; Kim & Lewis 2014; Kuipers von Lande & Worth 2010). Thus, the CORA method was adopted from human surgery and used to define the sagittal plane deformity and plan the corrective ostectomy. Using this method, we aimed to achieve surgical correction of the sagittal plane angulation on the right so that, post-operatively, it had a similar anatomical alignment to the normal left femur. Normal breed reference values for sagittal plane deformities should be investigated as they are useful for cases with bilateral femoral deformities.

A closing wedge ostectomy was chosen to allow circumferential bone-on-bone contact at the ostectomy site. This allowed load sharing of the bone-implant construct and minimised the chance of catastrophic failure of the surgical implant. Additionally, it was determined that a relatively small wedge of bone would be removed to correct the moderate sagittal plane deformity in this case. Thus, the discrepancy in femoral length would not be exacerbated. An opening wedge osteotomy is technically more challenging, places more strain on the surgical implants and is typically reserved for cases with severe femoral shortening or angulation (Fox & Tomlinson 2012; Kim & Lewis 2014).

One of the concerns in this case would be iatrogenic translation at the ostectomy site. This may occur because of the completion of the ostectomy and angulation correction axis (‘hinge point’) at a level different to the CORA (Fox & Tomlinson 2012). In the current case, it was necessary to perform the ostectomy at a different level from the determined CORA in order to allow an adequate size of the distal fragment for placement of a 3.5-mm supracondylar bone plate. In future, it might be better to apply Paley’s second rule of osteotomies where the anatomical alignment of the femur is maintained by translation of one of the bone segments (Fox & Tomlinson 2012).

In conclusion, we advocate surgical correction of sagittal plane deformities of the distal femur using the CORA method. Concurrent physiotherapy in the post-operative period limits complications such as quadriceps contracture, addresses muscular atrophy and fibrosis of the joint capsule. In this case, the functional extension of the right stifle was improved, allowing weight-bearing on the operated limb. Overall, a good functional outcome was achieved, which is consistent with previously reported cases with similar deformities (Kim & Lewis 2014; Kuipers von Lande & Worth 2010).
